# Dr. W. H. R. Rivers

**Published:** 1922-07

**Authors:** 


### OBITUARY.

### DR. W. H. R. RIVERS.

We regret to record the death, at the age of 56 years, after a short illness, of Dr. W. H. R. Rivers, F.R.S., Fellow and Prælector in Natural Sciences, St. John's College, Cambridge, and President of the Anthropological Society. In our May issue we reviewed at length his great work, "Instinct and the Unconscious." Since the death of Hughlings Jackson no such philosophical intellect has illuminated the problems of mental disorder. He was also a distinguished anthropologist. His "History of the Melanesian Society" is a model of original research, in which he reconstructed the history of the migrations of the Melanesians from their culture. His doctrine that hysteria is essentially protective may be taken as a striking example of his work. It was based on a study of the mental breakdown of soldiers under the stress of war. He found that the symptoms of hysteria which appeared had "the common feature that they unfit their subject for further participation in warfare." They appeared in order to "protect" the individual.

